# Pilot evaluation of a compact targeted next-generation sequencing with minimum biocontainment for rapid diagnosis of drug-resistant tuberculosis

**DOI:** 10.1128/spectrum.02917-25

**Published:** 2026-05-18

**Authors:** Radha Gopalaswamy, Ashok Kumar Shanmugavel, Bhargavi Subramanian, Naveen Kumar Nagarajan, Ashok Selvaraj, Adhin Bhaskar, Manisha Parthasarathy, M. Michel Prem Kumar, P. Sivaraman, Chandrasekaran Padmapriyadarsini, Sivakumar Shanmugam

**Affiliations:** 1 ICMR - National Institute for Research in Tuberculosis 29888 , Chennai, India; 2 SRM Institute of Science and Technology 599504 , Chennai, India; 3 Vellore Institute of Technology 30026 , Vellore, India; Rutgers New Jersey Medical School, Newark, New Jersey, USA

**Keywords:** targeted sequencing, Trueprep AUTO device, BPaLM regimen, resistance profiling, RR/MDR-TB, Oxford nanopore technology, Deeplex Myc-TB

## Abstract

Next-generation sequencing is a more rapid and robust technique than phenotypic drug susceptibility testing (pDST) for diagnosing drug-resistant tuberculosis (DR-TB). Targeted next-generation sequencing (tNGS) using Oxford Nanopore Technology (ONT) is an easy, tabletop, and portable option for comprehensive drug resistance profiling. Sputum sample/*Mycobacterium tuberculosis* (*M. tb*) isolates from 351 bacteriologically confirmed TB patients between January 2022 and June 2023 were used in the study. We assessed the feasibility of using DNA isolated with an extraction device from a routine rapid point-of-care test, thereby requiring minimal biocontainment. A commercial Deeplex Myc-TB primer set was used with *M. tb* for tNGS. Library preparation was done with a rapid barcoding kit from ONT, and run using MinION Mk1C (ONT). tNGS performed with the DNA extracted using the Trueprep device showed successful runs with lineage and DR prediction for 252 samples. As part of this study, a bioinformatics pipeline was customized in-house and validated at ICMR-NIRT and has been made publicly available on GitHub. Among the runs, the highest depth was obtained for *inhA* (1160.11), while *rrl* (89.52) had the lowest depth. tNGS showed comparable sensitivity and specificity with a sensitivity of 95% for RIF, 88% for INH, and 100% for FQ, AMG, and LZD. The agreement between tNGS and pDST by Cohen’s kappa was between 0.90 and 1.00 for RIF, FQ, AMG, STR, and LZD, and less than 0.8 for PZA and ETH. Our pilot evaluation study was conducted to assess the feasibility of implementing tNGS using an ONT device from direct unprocessed sputum using a Trueprep AUTO extraction device for DNA extraction independent of a BSL3 or TB containment laboratory. A conceptual diagnostic algorithm for low- and middle-income country to perform tNGS as a single follow-on molecular test under programmatic setting, together with an in-house bioinformatic pipeline, to ensure equity access to rapid DR-TB testing has been proposed.

**IMPORTANCE:**

Our study highlights the importance of using point-of-care test DNA directly for targeted next-generation sequencing (tNGS), thus involving minimum biocontainment. The testing is rapid and promises tNGS in different tiers of tuberculosis laboratory under programmatic setting in low-resource high-burden setting.

## INTRODUCTION

In 2023, an estimated 10.8 million people fell ill with tuberculosis (TB), with 8.2 million newly diagnosed cases of TB globally. Despite increased diagnosis and treatment, an estimated 1.25 million deaths were caused by TB in 2023. During this period, rifampicin resistance testing was done only for 79% of bacteriologically confirmed TB cases, with 5.5% detected as extensively drug-resistant (XDR) and pre-XDR TB (1). Hence, there is an augmented need for drug-resistant (DR)-TB testing among bacteriologically confirmed TB cases and their timely enrollment into appropriate DR-TB treatment regimens. Rapid diagnosis of DR-TB with comprehensive drug resistance profiling is required to initiate an appropriate treatment regimen (2, 3). While low and moderate complexity nucleic acid amplification technologies (NAATs) enable rapid detection of resistance to key first- and second-line TB drugs, they fail to cover other essential drugs like pyrazinamide (PZA), ethambutol (EMB), bedaquiline (BDQ), linezolid (LZD), clofazimine (CFZ), delamanid (DLM), and pretomanid (PTM). Phenotypic drug susceptibility testing (pDST) remains the benchmark for detecting DR patterns in these drugs, although this method has an overall turnaround time of 5–6 weeks (2). As an alternative, next-generation sequencing (NGS) with various platforms became available in higher reference laboratories to detect DR-TB cases. NGS includes whole-genome sequencing (WGS) and targeted next-generation sequencing (tNGS) to identify single-nucleotide polymorphisms (SNPs) in genes conferring DR (4). Direct sequencing of sputum samples poses a significant challenge due to low yields of *M. tb* DNA, necessitating the need for culture to proceed to WGS (5). Besides, WGS is expensive, requires a sophisticated laboratory setup and complex bioinformatics infrastructure, and cannot be widely utilized in low-resource settings (6, 7). The implementation of a unique, hand-held sequencing system like MinION (Oxford Nanopore technology) in high TB burden and low-resource settings proves its use as a clinical diagnostic tool. It is portable, robust, and cost-effective and could conceivably be used in near-patient settings to revolutionize TB DST and the treatment decision-making process (8, 9). Deeplex Myc-TB master mix (Genoscreen) endorsed by WHO covers 18 regions in the *M. tb* genome that are associated with drug resistance (*rpo*B, *fab*G1, *kat*G, *rps*L, *rrl*, *inh*A, *ahp*C, *pnc*A, *gyr*B, *gyr*A, *eth*A, *eis*, *emb*B, *rrs, tly*A, *rpl*C, *gid*B, and rv0678). It enables species identification and genotyping of *M. tb* (10, 11). WHO recommends Deeplex Myc-TB for DR profiling against RIF, isoniazid (INH), fluoroquinolones (FQ), amikacin (AMK), streptomycin (STR), ethionamide (ETH), PZA, BDQ, LZD, EMB, and CFZ (2, 12). Previous studies on Deeplex Myc-TB with patient samples have identified first- and second-line DR with a sensitivity of 95.3% and specificity of 97.4% (13–20). The DR-TB regimen for MDR/RR-TB is currently offering 6–9 months of bedaquiline, pretomanid, linezolid, and moxifloxacin (BPaLM) or BPaL regimen warranting rapid and more expansive DR profiling for every DR-TB patient (2, 3).

This pilot evaluation study aims to understand the implementation feasibility of the tNGS-based rapid DR-TB detection with a compact, low-cost Oxford Nanopore Technologies integrating it with an automated DNA extraction device, Trueprep AUTO, under a programmatic setting. This extraction device is validated as part of the Truenat MTB POCT kit under the National TB Elimination Program (NTEP). The Trueprep AUTO device is portable, cost-effective, automated, battery-operated, and works in ambient temperature, with no requirement for sophisticated equipment like air conditioning. When used in peripheral settings, it enables DNA extraction from sputum samples through a simple liquefaction and lysis system, without requiring a biosafety cabinet.

Under the NTEP in India, initial tests for presumptive TB patients are performed using low complexity NAATs like Truenat MTB and GeneXpert MTB/RIF for the detection of TB. Following the detection of TB, follow-up testing on presumptive DR-TB patients is done using a line probe assay (LPA) and pDST by the MGIT (21). Hence, Trueprep DNA, Genolyse DNA, and MGIT cultures would be available in NTEP labs as part of diagnosis and follow-up testing for additional DR, particularly in BPaLM or BPaL patients. Though solid culture by LJ is not part of the routine NTEP algorithm, it is selectively used to extract DNA for WGS. The study aims to evaluate the use of Trueprep AUTO for DNA extraction from unprocessed sputum under programmatic settings in tNGS with minimal or no biocontainment.

## MATERIALS AND METHODS

### Study population and setting

**Fig 1:**
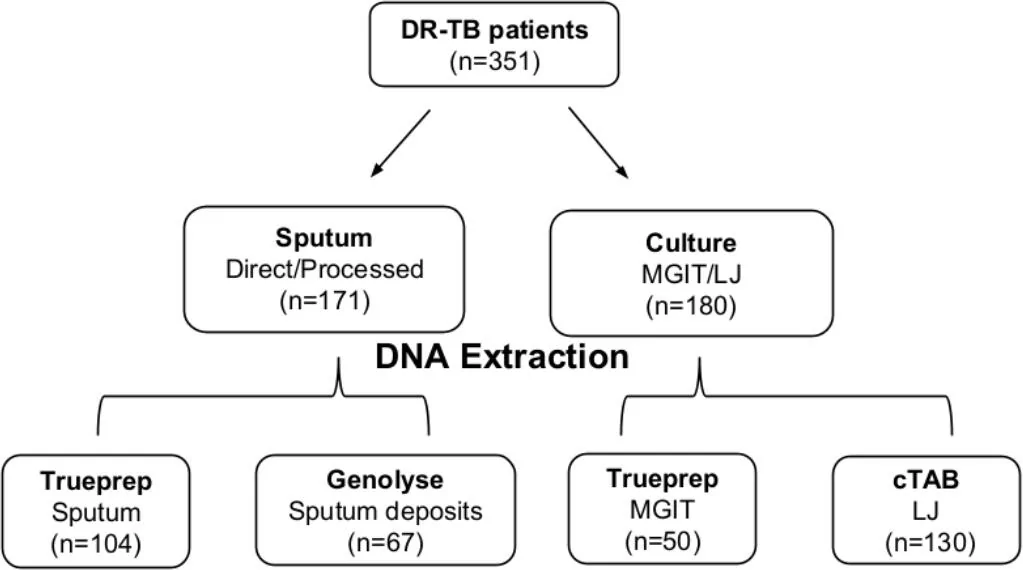
Sample type and method of DNA extraction. Samples tested using tNGS include direct or processed sputum and cultures as indicated. DNA extraction was done using the Trueprep and Genolyse methods from direct sputum and processed sputum deposits, respectively. Trueprep and conventional extraction methods were used for MGIT and LJ cultures, respectively.

Sample types from 351 patients with bacteriologically confirmed pulmonary TB from DR-TB centers of Tamil Nadu state, transported to the Indian Council of Medical Research-National Institute for Research in Tuberculosis (ICMR-NIRT), were included. At ICMR-NIRT, samples are tested for DR using first- and second-line LPA, followed by pDST using culture, as per the national guidelines (21). In this study, we used archived *M. tb* samples collected between January 2022 and June 2023 and freshly extracted DNA using different methods at ICMR-NIRT. Ethics approval for the study was obtained from the NIRT Institutional Ethics Committee (IEC No. 2022 003). Sample types include sputum samples, processed sputum deposits, and *M. tb* cultures grown in BACTEC Mycobacterial growth indicator tube (MGIT) and Löwenstein–Jensen (LJ) medium (Fig. 1). Inclusion criteria included smear-positive direct or processed sputum and/or culture positive for *M. tb* by liquid or solid media. Exclusion criteria included smear-negative direct or processed sputum and/or culture-negative for *M. tb*.

### Processing of sputum and culturing of *M. tb*

Sputum samples were processed by the NalC-NaOH method, and the deposits were resuspended in PBS solution and used for downstream purposes (22). Sputum smears are prepared using sputum or processed deposits, stained for fluorescence microscopy, and graded according to the IUATLD/WHO scale (22). The processed sputum deposits were inoculated into in-house LJ slants (custom made at ICMR-NIRT) and BACTEC MGIT 960 tubes (BD Biosciences) and incubated at 37°C for growth (22). As recommended by the WHO, pDST was performed on MGIT 960 tubes for various drugs at their critical concentration (23). All the chemicals were purchased from Sigma Aldrich for the preparation of reagents.

### Extraction of *M. tb* genomic DNA

For comparison, *M. tb* genomic DNA was extracted using different methods, including the conventional Cetyltrimethylammonium bromide (cTAB) method. The methods included extraction procedures that could be performed with and without the requirement of a BSL3 facility. For sputum samples, DNA extraction was carried out using the Trueprep AUTO device (MolBio Diagnostics) for direct sputum (referred to as Trueprep sputum) and Genolyse solution (Hains Life Sciences) for processed sputum deposits (referred to as Genolyse sputum), respectively. The Trueprep AUTO device was also used for the MGIT cultures (referred to as Trueprep MGIT). The conventional cTAB-NaCl method was used to extract *M. tb* genomic DNA (referred to as cTAB-LJ) from LJ slants as previously described (24). All purified DNA was resuspended in molecular-grade water and stored at −20°C until ready to use. Initial DNA quantification was done using Nanodrop (Thermo Fisher Scientific), and the concentration readings (ng/µL) and the purity of the sample, namely, 260/280 and 260/230, were recorded. Qubit HS dsDNA Quantification Assay Kits (Thermo Fisher Scientific) were used with a Qubit Fluorometer (Thermo Fisher Scientific) for quantitation of DNA for normalization during the NGS runs.

### Line probe assay

LPA was routinely performed using MTBDR*plus* and MTBDR*sl* (Hains Life Sciences) for bacteriologicallt confirmed TB patients as previously described (25, 26). The interpretation and reporting of DR were done using the GLI guidelines (27).

### Targeted NGS

An equal quantity of input DNA (50 ng) was used across every run, and the amplification of targets was done using a master mix supplied with the Deeplex Myc-TB kit (Genoscreen). PCR clean-up and size selection were done using AMPure beads (Beckman Coulter) as instructed and purified using a magnetic rack (New England Biolabs). Rapid barcoding and adapter ligation were done for the library preparation using SQK-RBK110.96 per the manufacturer’s instructions. MinION Mk1C, flow cell R10.3, was primed and loaded with the library for the sequencing run.

### Whole-genome sequencing

For WGS, library preparation was done using the Nextera XT DNA Library Preparation Kit (Illumina). The samples were indexed using the Nextera XT Index Kit (Illumina). Sequencing was performed in MiSeq using the MiSeq Reagent Kit v2 600 Cycles (Illumina). Library preparation and bioinformatic analysis using NIRT Camspred pipeline for DR prediction were done as previously described (28–30).

### Bioinformatic analysis for tNGS

Using the in-house bioinformatics pipeline, MinION sequencing reads were basecalled using Albacore and demultiplexed using Guppy. ONT reads ≥0.9 probabilistic confidence scores were selected and merged into a single FASTQ file and mapped to the *M. tb* H37Rv reference genome (NC_000962.3) using Minimap2 v2.17-r954-dirty with the -ax map-ont option. SAM files were converted into sorted BAM files using Samtools v1.9, and variants were called using Bcftools v1.9 mpileup with default parameters to generate VCF files. Variants were filtered using an in-house Python script based on base quality, mapping quality, read depth, and allele frequency. For initial variant filtering, only variants with base quality ≥30, mapping quality ≥30, and read depth ≥5 were retained. Given the differences in quality scoring between Oxford Nanopore and Illumina sequencing, the QUAL score was used as a supporting metric rather than a primary filtering criterion as previously described (31). Following initial variant quality filtering, retained variants were classified based on alternate read support, strand balance, and total sequencing depth. Mutations were defined as fixed DR variants when ≥90% of reads supported the alternate allele, with a minimum read depth of ≥20 and bidirectional strand support. Variants were classified as heteroresistant when alternate read support was ≥15% and <90% of total reads, with a minimum depth of ≥20 and bidirectional strand support for both reference and alternate alleles (31). DR prediction and annotation were performed by comparing filtered variants against the Indian mutation catalog V2.0 (32).

### Phenotypic DST

pDST was performed using MGIT 960 (BD Biosciences) for various drugs at their critical concentrations as per the WHO guidelines (23).

### Statistical analysis

The sensitivity, specificity, and accuracy were estimated with 95% confidence level (CI). In addition, the positive predictive value and negative predictive value were calculated. The agreement between the phenotypic and genotypic DST results was calculated using Cohen’s kappa and concordance at 95% CI values. Kappa values of 0.81–1.00 indicated almost perfect agreement, while the values of 0.61–0.80 indicated substantial agreement. All the analysis was carried out using the R software version 4.5.2

## RESULTS

During the study period between January 2022 and June 2023, 351 samples from bacteriogically confirmed TB patients were tested by tNGS on a MinION using Deeplex Myc-TB primers.

### Sample distribution and comparison of DNA extraction methods

**Fig 2:**
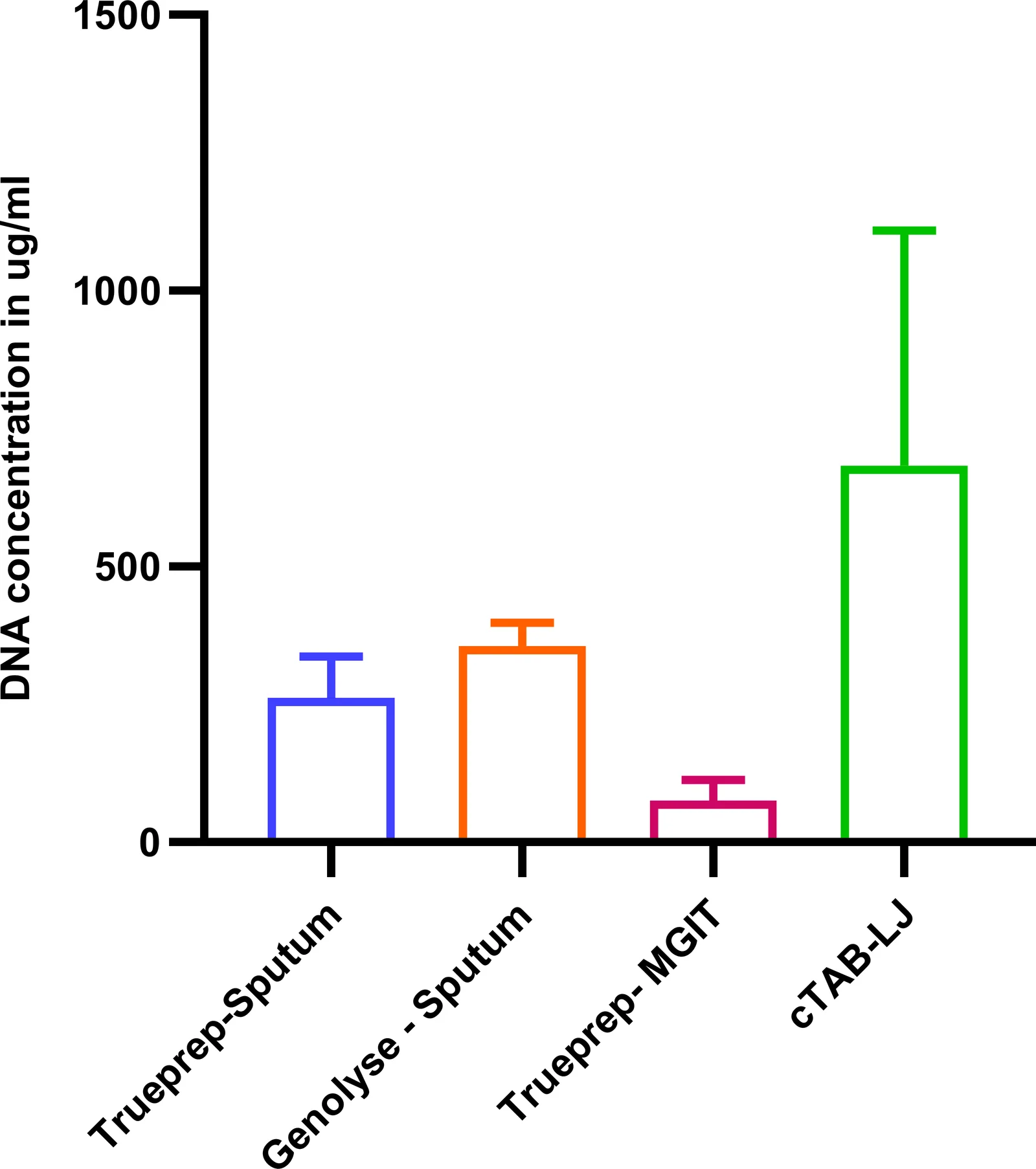
Yield of DNA across different methods of extraction. Distribution of DNA quantities extracted from sputum and culture by Trueprep DNA in comparison to the alternative method. The X-axis describes DNA extracted across different methods of extraction, along with the respective sample type used for extraction. The Y-axis indicates the median concentration of DNA (μg/mL) with IQR.

The samples included sputum (171 samples) as well as *M. tb* cultures (180 cultures from MGIT or LJ) from bacteriologically confirmed pulmonary TB patients (Fig. 2). Runs included DNA samples extracted using the Trueprep AUTO device and compared with DNA extracted routinely using other extraction methods from sputum or cultures under the NTEP at the study site (Fig. 1).

Among the sputum samples, the average quantity of DNA extracted from the Trueprep AUTO device (311.82 μg/mL) and the Genolyse method (291.77 μg/mL) was comparable. At the same time, from culture, it was observed that the cTAB extraction method gave a superior yield (743.61 μg/mL) compared to the Trueprep method (86.67 μg/mL). Distribution of DNA quantity as median with IQR among the different extraction methods indicated a diverse range of concentrations (Fig. 2). However, all the samples were normalized before the setup of polymerase chain reaction (PCR) to ensure an equimolar amount of the template DNA across all samples.

### Comparison of different DNA types with tNGS runs

**Fig 3:**
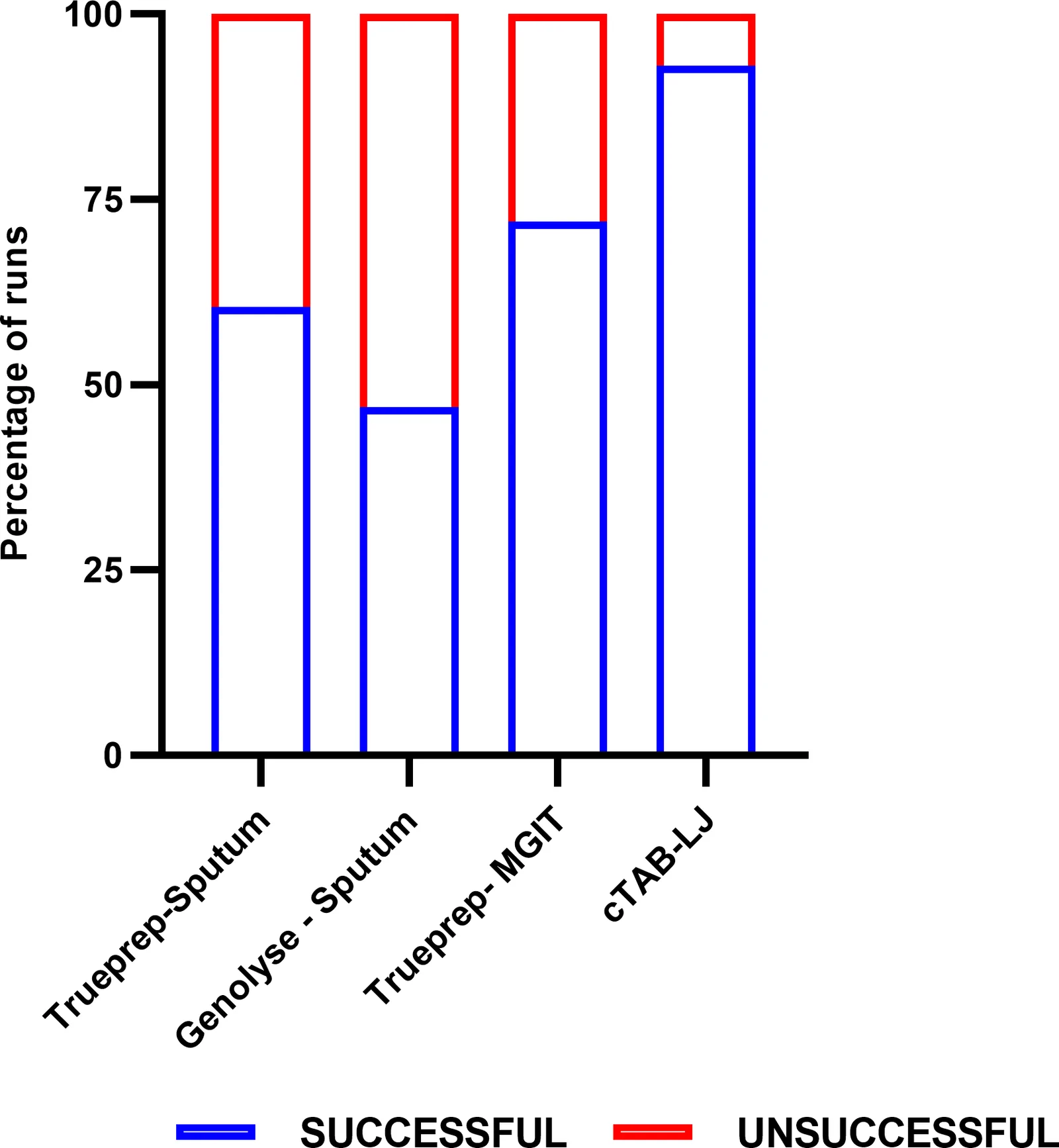
Comparison of successful tNGS runs among the different sample types and method of DNA extraction in percentage of the total. The X-axis describes the different methods of extraction along with the respective sample type used for extraction. The Y-axis indicates the percentage of tNGS runs, with an individual bar graph describing the proportion of successful and unsuccessful runs.

A successful tNGS run was assessed based on the output, including the coverage and depth, and not exclusively on the variant calling. Upon comparison of the DNA extracted from Genolyse and Trueprep DNA for direct tNGS from sputum samples, we observed that Trueprep DNA gave 60.58% successful runs compared to Genolyse DNA, which was 47.7% (Fig. 3). Among the DNA extracted from *M. tb* cultures, we found 93.8% yielded successful runs with the cTAB method of DNA extraction (LJ slope cultures) and 72.0% from the Trueprep method of DNA extraction (MGIT cultures) (Fig. 3). Among the culture, cTAB method from LJ culture yielded better results, while for direct sputum, we observed that the Trueprep method yields better results.

### Bacillary load and successful tNGS runs

Smear acid-fast bacilli (AFB)-positive samples (AFB grades 1+, 2+, and 3+) were used in this tNGS study (predominantly 2+), and no significant differences were observed between the different smear grades for sputum samples. The percentage of successful runs for the overall sputum samples was 58.58% (Fig. S1). Among the direct sputum, we observed a similar number of successful tNGS runs among the bacillary load ascertained as colony-forming units per milliliter (CFU/mL) since 90% of the samples had a CFU/mL value of 10^5^–10^7^ (Fig. S2).

### Customized bioinformatic pipeline for tNGS using MinION

**Fig 4:**
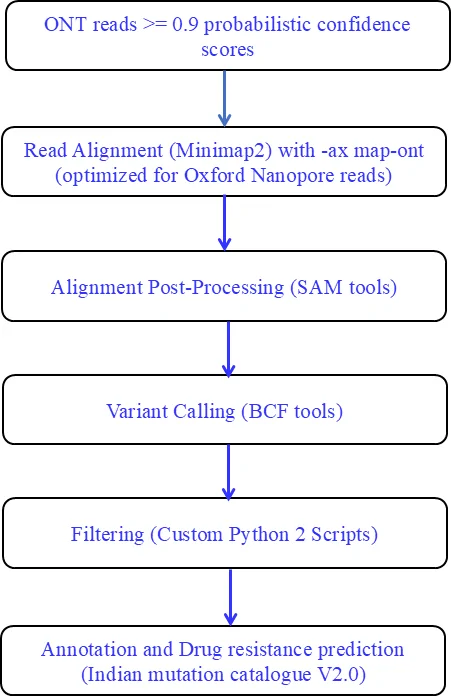
Consort style diagram of the tNGS bioinformatic pipeline. Using the in-house bioinformatics pipeline, MinION sequencing reads were basecalled using Albacore and demultiplexed using Guppy. Reads from each sample were merged into a single FASTQ file and mapped to the *M. tb* H37Rv reference genome (NC_000962.3) using Minimap2 v2.17-r954-dirty with the -ax map-ont option. SAM files were converted into sorted BAM files using Samtools v1.9, and variants were called using Bcftools v1.9 mpileup with default parameters to generate VCF files. Variants were filtered using an in-house Python script based on base quality, mapping quality, read depth, and allele frequency. Drug resistance prediction and annotation were performed by comparing filtered variants against the Indian mutation catalog V2.

In this study, we successfully customized an in-house tNGS pipeline (Fig. 4), which is written in shell scripts, enabling faster execution of the pipeline. The pipeline uses Minimap2 software for variant calling. High-quality variances were filtered based on the parameters described in the Materials and Methods section. DR prediction and annotation were performed by comparing filtered variants against the Indian mutation catalog V2.0, validated to be statistically similar to WHO mutation catalog ver2 (32). Sequencing reads obtained for targeted NGS using MinION Mk1C are available at the ICMR-NIRT Chennai central server. The in-house tNGS pipeline has been provided in the following link (https://github.com/shanmugamsiva/tNGS).

### tNGS analysis for lineage and SNPs

**Fig 5:**
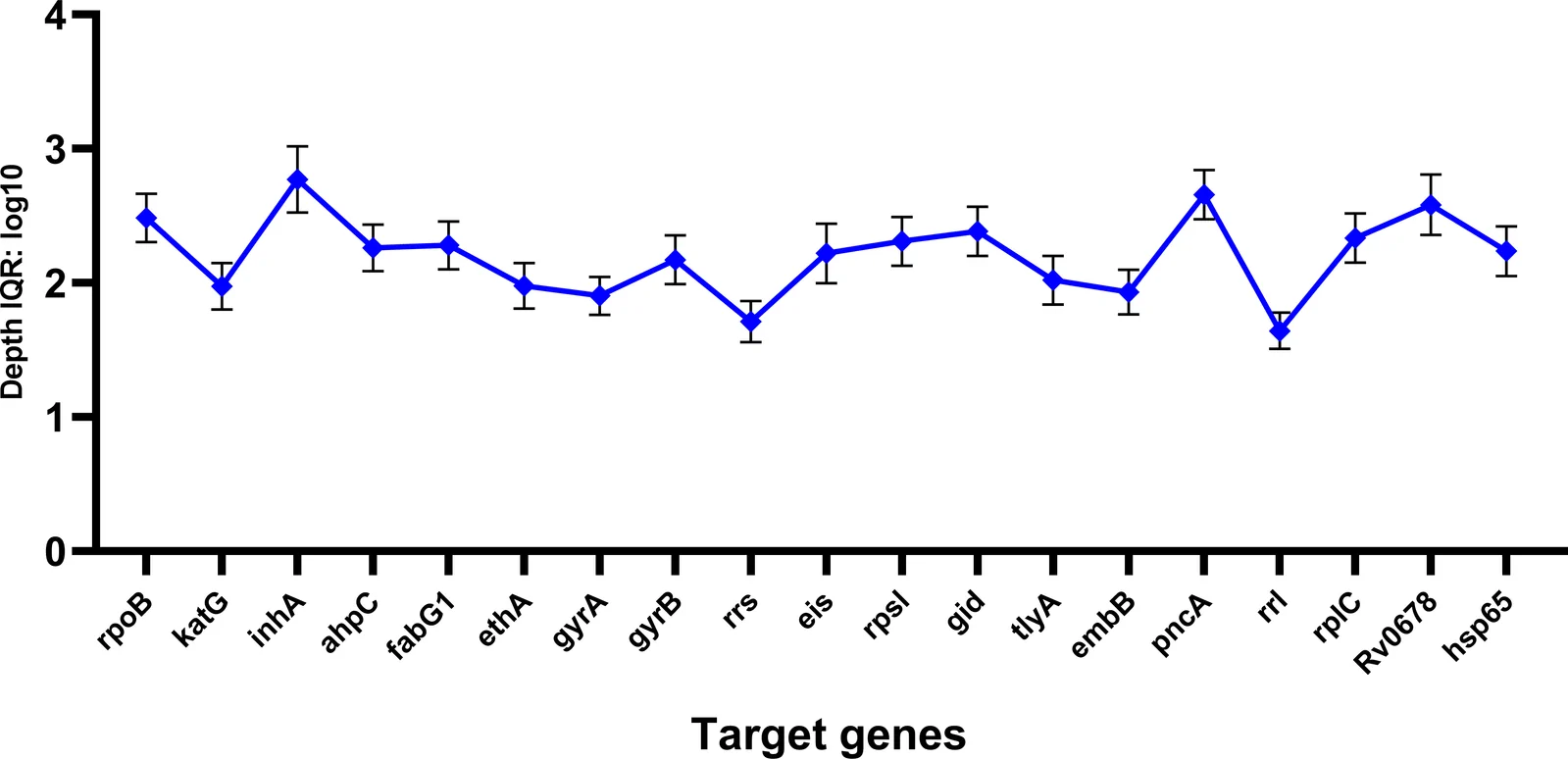
Depth across successful runs. The figure illustrates the distribution of sequencing depth across all genes in the clinical samples analyzed in this study. The X-axis represents the genes, while the Y-axis indicates their corresponding log10 of depth at their IQR.

The SNPs for all the mutations were analyzed, and we observed S315T in *katG* as the predominant mutation for INH, and S450L in *rpoB* for RIF. The SNPs identified for different DR genes were the most common among previously published studies (Table 1). The depth of all genes present in Deeplex Myc-TB primers was analyzed (Fig. 5). The depth was calculated as the mean and IQR among all the runs done using MinION, Oxford Nanopore Technology (Table S1). The highest depth was obtained for *inhA* (1160.11), followed by *pncA* (916.37). In contrast, *rrl* (89.52), followed by *rrs* (101.08), has the lowest depth among the runs (Fig. 5). The distribution of SNPs varies among different genes and indicates some are conserved. In contrast, others are distributed to different regions of the gene. The *rpoB* gene for RIF is highly conserved, while *pncA* shows a distribution of various SNPs (Fig. S3).

**TABLE 1:** Common SNP for different DR genes^*a*^

Drugs	Resistance gene(s)	Function	Common SNPs	Frequency (n)	Total resistant (N)	Percentage
RIF	*rpoB*	RNA polymerase	S/450/L	65	98	66.33
INH	*katG*	Catalase peroxidase	S/315/T	80	101	79.21
	*p-fabG1: inhA*	Enoyl-ACP reductase promoter	C/−15/T*	30	101	29.70
ETH	*p-fabG1: inhA*	Enoyl-ACP reductase promoter	C/−15/T*	21	30	70.00
FQ	*gyrA*	DNA gyrase subunit A	D/94/G	23	71	32.39
AMG	*rrs*	16S ribosomal RNA	A/1401/G*	17	52	32.69
Streptomycin	*rpsL*	Ribosomal protein S12	K/43/R	42	55	76.36
Ethambutol	*embB*	Arabinosyl transferase	M/306/V	22	64	34.38
Pyrazinamide	*pncA*	Pyrazinamidase	G/132/A	6	45	13.33
Linezolid	*rplC*	Ribosomal protein L3	C/154/R	8	8	100.00

^*a*^ The most common SNP for every drug, along with their frequency, total DR per drug class, and percentage, is given. * indicates changes in nucleotides, while the others indicate changes in amino acids at the respective position as indicated by numbers.

Sequencing depth and coverage are two crucial concepts in the field of genomics, especially when discussing NGS. This is particularly important in tNGS, as the target genes need to be amplified to obtain a sufficient quantity (the depth) for predicting DR. The percentage coverage with its mean, SD, and CV was calculated for the runs (Table S2). Coverage describes the gene length covered, including the hotspots of mutation. In our study, the coverage was almost 100% for *ethA, gid, rpsL, pncA, rrs, tlyA*, Rv0678*, rrl*, and the rest varied from 10% to 100%. We called the mutation when the coverage was >60%. The depth varied between the genes, but the mean was around 1,000×. We did not call the resistance for a drug if the gene is less than >50×.

**Fig 6:**
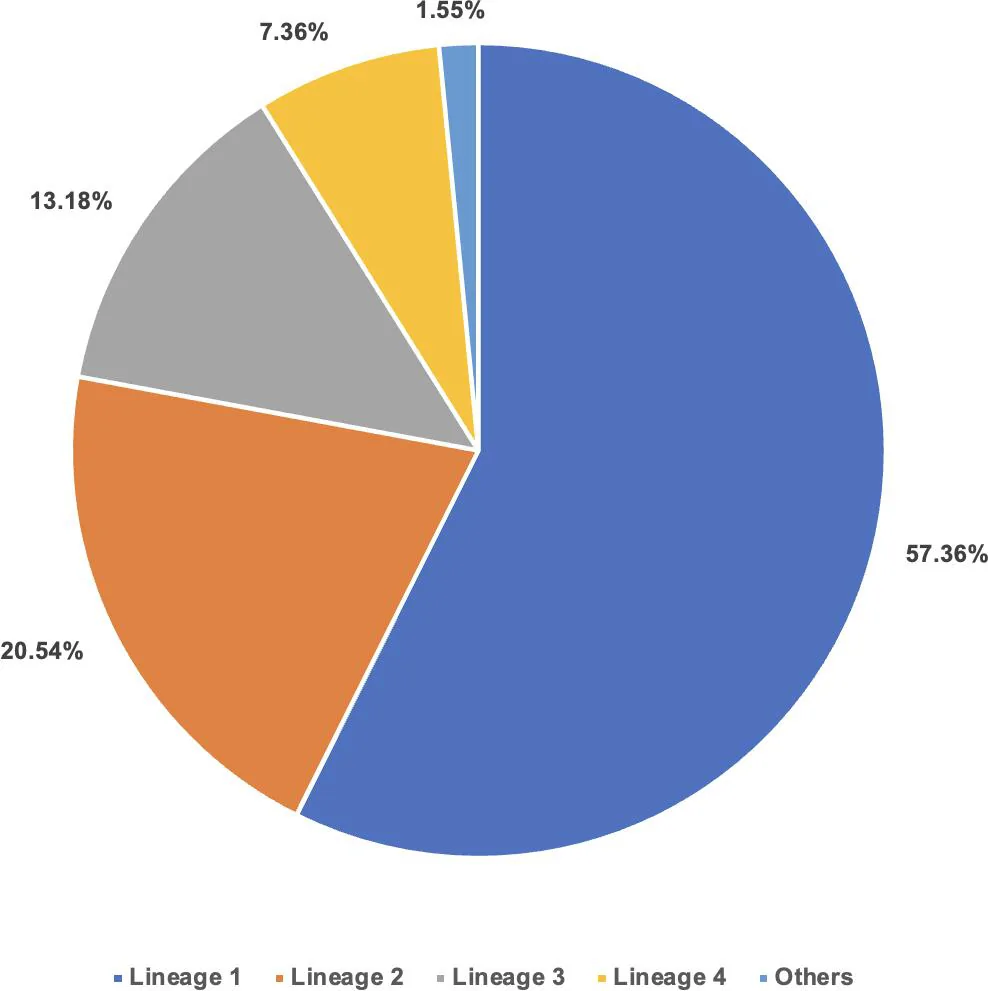
Distribution of different *M. tb* lineages. The pie chart indicates the percentage distribution of lineages 1, 2, 3, 4, and the others.

The *M. tb* phylogenetic lineage distribution among the tNGS samples with successful runs was analyzed and tabulated (Fig. 6). Lineage 1 was observed in the majority (57.36%) of the samples, and the least was lineage 4 (7.36%).

**Fig 7:**
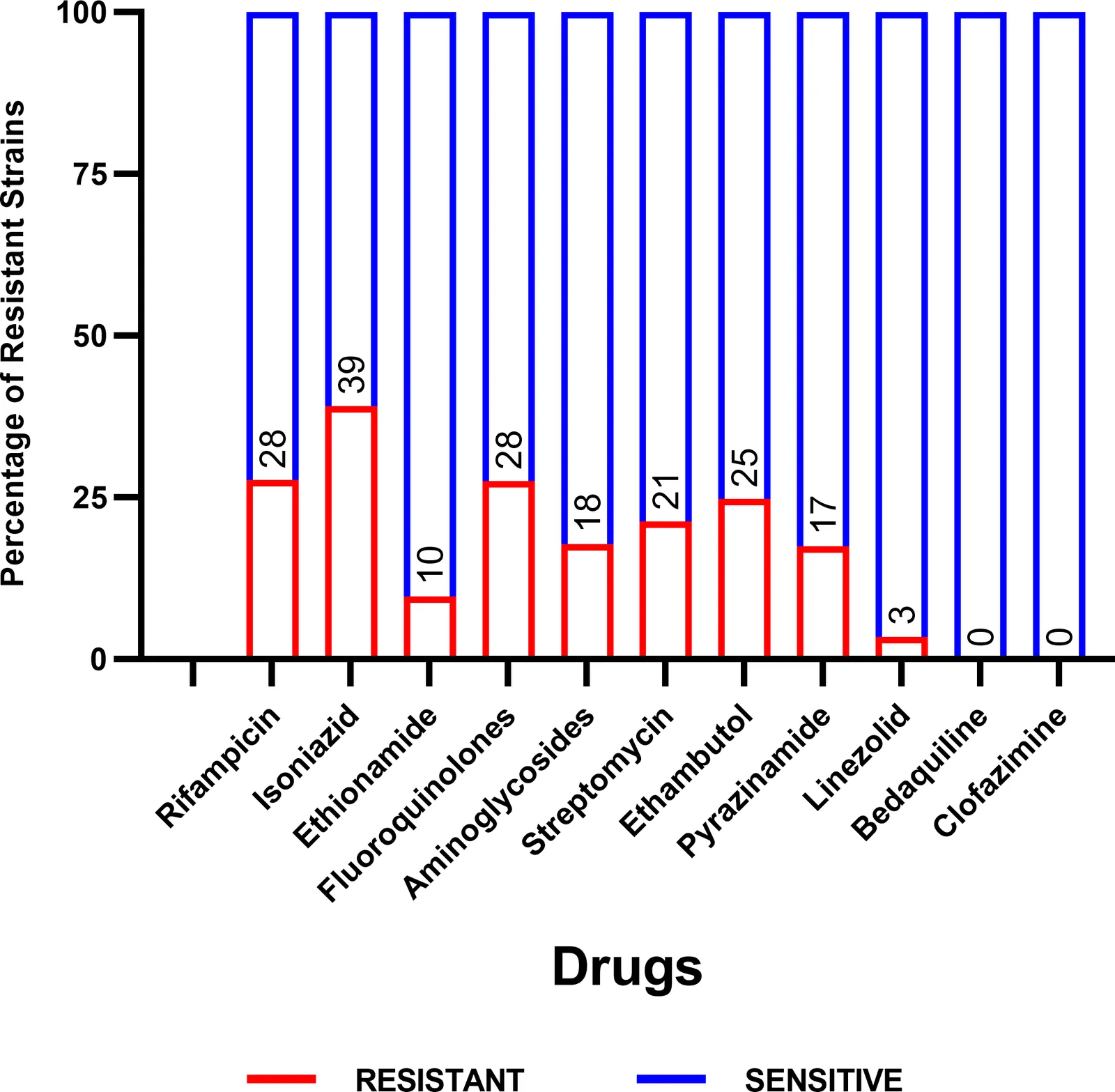
Drug resistance profile among samples. Distribution of resistance to anti-TB drugs as observed among the tNGS samples grouped into drug class. The X-axis indicates the drugs, and the Y-axis indicates the total percentage of resistant samples against each drug.

The resistance distribution to various drugs has been analyzed (Fig. 7). The 252 samples that showed successful runs in the tNGS assay had varied resistance to the first-, second-line, and other anti-TB drugs. Among the first-line drugs, 39% were resistant to INH, while 28.0% showed resistance to RIF, followed by EMB (25.0%) and PZA (17.0%). Among second-line medications, 28.0% showed resistance to FQ(s), followed by 18.0% to aminoglycosides (AMG). The rest of the mutations were distributed among STR, ETH, and LZD. No resistance to BDQ or CFZ was observed using tNGS among the study samples.

### Comparison of tNGS runs with LPA, WGS, and pDST data

The first- and second-line DR was compared with first- and second-line LPA results for 84 samples, and a 99% concordance was observed between these two results (data not shown). The LPA, tNGS, and WGS were compared with pDST results wherever available as indicated (Table 2). The data were compared for lineage and DR profiling. Lineage data from overall tNGS showed a 97.5% concordance with the WGS data. tNGS showed a sensitivity of 95.0% (95% CI: 83.0–99.0) for RIF and 88.0% (74.0–96.0) for INH, which was comparable to WGS. However, tNGS showed superior sensitivity for FQ at 100% (95% CI: 89.0–100) and AMG having 100% (95% CI: 82.0–100). LZD, BDQ, and CFZ showed 100% specificity with tNGS which was comparable to WGS when pDST was used as a reference standard. All eight strains that showed a positive result for LZD resistance in tNGS also displayed resistance to LZD in pDST (1 µg/mL). Overall, the agreement between tNGS and pDST assessed by Cohen’s kappa was 1.00 for FQs, AMG, and LZD, and above 0.85 for RIF, INH, EMB, and PZA, all of which indicated perfect agreement.

**TABLE 2:** Performance characteristics of tNGS compared to WGS and LPA^*a*^

Test	Drug	Profile	pDST		Performance (95% CI)					
			R	S	Sensitivity	Specificity	PPV	NPV	Concordance	Cohen's kappa
tNGS	RIF	R	37	2	95.0 (83.0–99.0)	97.0 (89.0–100)	95.0 (83.0–99.0)	97.0 (89.0–100)	96.0 (90.0–99.0)	0.92 (95% CI: 0.84–0.92)
		S	2	63						
WGS	RIF	R	36	1	97.0 (86.0–100)	99.0 (92.0–100)	97.0 (86.0–100)	99.0 (92.0–100)	98.0 (93.0–100)	0.96 (95% CI: 90.0–0.96)
		S	1	66						
LPA	RIF	R	19	1	100 (82.0–100)	93.0 (0.68–100)	95.0 (75.0–100)	100 (77.0–100)	97.0 (85.0–100)	0.94 (95% CI: 0.82–0.94)
		S	0	14						
tNGS	INH	R	37	1	88.0 (74.0–96.0)	98.0 (91.0–100)	97.0 (86.0–100)	92.0 (83.0–97.0)	94.0 (88.0–98.0)	0.88 (95% CI: 78.0–88.0)
		S	5	61						
WGS	INH	R	37	2	92.0 (80.0–98.0)	97.0 (89.0–100)	95.0 (83.0–99.0)	95.0 (0.87–99.0)	95.0 (89.0–98.0)	0.90 (95% CI: 0.81–90.0)
		S	3	62						
LPA	INH	R	16	1	89.0 (65.0–99.0)	94.0 (70.0–100)	94.0 (71.0–100)	88.0 (64.0–99.0)	91.0 (76.0–98.0)	0.82 (95% CI: 63.0–0.82)
		S	2	15						
tNGS	FQ	R	32	0	100 (89.0–100)	100 (95.0–100)	100 (89.0–100)	100 (95.0–100)	100 (96.0–100)	1.00 (95% CI: 1.00–1.00)
		S	0	71						
WGS	FQ	R	30	0	100 (88.0–100)	100 (95.0–100)	100 (88.0–100)	100 (95.0–100)	98.0 (93.0–100)	1.00 (95% CI: 1.00–1.00)
		S	0	73						
LPA	FQ	R	17	0	100 (80.0–100)	100 (79.0–100)	100 (80.0–100)	100 (79.0–100)	100 (89.0–100)	1.00 (95% CI: 1.00–1.00)
		S	0	16						
tNGS	AMG	R	19	0	100 (82.0–100)	100 (96.0–100)	100 (82.0–100)	100 (96.0–100)	100 (96.0–100)	1.00 (95% CI: 1.00–1.00)
		S	0	84						
WGS	AMG	R	17	0	94.0 (0.73–100)	100 (96.0–100)	100 (80.0–100)	99.0 (94.0–100)	99.0 (95.0–100)	0.97 (95% CI: 90.0–0.97)
		S	1	85						
LPA	AMG	R	19	1	100 (75.0–100)	95.0 (75.0–100)	93.0 (66.0–100)	100 (82.0–100)	97.0 (0.84–100)	0.94 (95% CI: 0.82–0.94)
		S	0	13						
tNGS	STM	R	20	0	95.0 (76.0–100)	100 (96.0–100)	100 (83.0–100)	99.0 (94.0–100)	99.0 (95.0–100)	0.97 (95% CI: 0.91–0.97)
		S	1	83						
WGS	STR	R	18	3	95.0 (74.0–100)	96.0 (90.0–99.0)	86.0 (64.0–97.0)	99.0 (93.0–100)	96.0 (90.0–99.0)	0.88 (95% CI: 76.0–88.0)
		S	1	82						
tNGS	EMB	R	0	0	NA	100 (94.0–100)	NA	100 (94.0–100)	100 (94.0–100)	NA
		S	0	56						
WGS	EMB	R	0	0	NA	97.0 (88.0–100)	NA	100 (94.0–100)	97.0 (88.0–100)	NA
		S	2	56						
tNGS	PZA	R	20	1	83.0 (63.0–95.0)	99.0 (93.0–100)	95.0 (76.0–100)	95.0 (88.0–99.0	95.0 (89.0–98.0)	0.88 (95% CI: 74.0–86.0)
		S	4	79						
WGS	PZA	R	20	2	87.0 (66.0–97.0)	98.0 (91.0–100)	91.0 (71.0–99.0)	96.0 (90.0–99.0)	95.0 (89.0–98.0)	0.86 (95% CI: 74.0–86.0)
		S	3	79						
tNGS	LZD	R	5	0	100 (48.0–100)	100 (96.0–100)	100 (48.0–100)	100 (96.0–100)	100 (97.0–100)	1.00 (95% CI: 1.00–1.00)
		S	0	102						
WGS	LZD	R	5	0	100 (48.0–100)	100 (96.0–100)	100 (48.0–100)	100 (96.0–100)	100 (96.0–100)	1.00 (95% CI: 1.00–1.00)
		S	0	98						
tNGS	CFZ	R	0	0	NA	100 (97.0–100)	NA	100 (97.0–100)	100 (97.0–100)	NA
		S	0	107						
WGS	CFZ	R	0	0	NA	100 (96.0–100)	NA	100 (96.0–100)	100 (96.0–100)	NA
		S	0	103						
tNGS	BDQ	R	0	0	NA	100 (96.0–100)	NA	98.0 (93.0–100)	98.0 (93.0–100)	NA
		S	2	103						
WGS	BDQ	R	2	0	100 (16.0–100)	100 (96.0–100)	100 (16.0–100)	100 (96.0–100)	100 (96.0–100)	1.00 (95% CI: 1.00–1.00)
		S	0	99						
tNGS	ETH	R	8	1	73.0 (39.0–94.0)	99.0 (94.0–100)	89.0 (52.0–100)	97.0 (91.0–99.0)	96.0 (90.0–99.0)	0.78 (95% CI: 0.57–78.0)
		S	3	91						
WGS	ETH	R	7	2	70.0 (35.0–93.0)	98.0 (92.0–100)	78.0 (40.0–97.0)	97.0 (91.0–99.0)	95.0 (89.0–98.0)	0.71 (95% CI: 0.47–71.0)
		S	3	91						

^*a*^ For samples with available pDST results, tNGS, WGS, and LPA results were assessed to study their sensitivity, specificity, positive predictive value, negative predictive value, concordance, and agreement Cohen’s kappa with 95% CI. tNGS—targeted next-generation sequencing; WGS—whole-genome sequencing; LPA—line probe assay; pDST—phenotypic drug susceptibility testing; RIF—rifampicin; INH—isoniazid; FQ—fluoroquinolone; AMG—aminoglycoside; STR—streptomycin; EMB—ethambutol; PZA—pyrazinamide; LZD—linezolid; CFZ—clofazimine; BDQ—bedaquiline; ETH— ethionamide; R—resistant; S—susceptible; PPV—positive predictive value; NPV—negative predictive value. CI—confidence interval; NA—not available.

Among the tNGS resistance prediction, two samples were discordant for RIF and one sample for AMG when tNGS was compared to LPA. Among the resistance profiling, three SNPs showed variations between WGS and tNGS—two samples showed one additional mutation in the *gyrB* and *gidB* genes each, and one sample showed a missing SNP in the *katG* gene. PZA showed discordance for five samples between tNGS and pDST. Two of the strains that were LZD resistant had additional BDQ/CFZ resistance in pDST but were not captured in the tNGS assay.

## DISCUSSION

Our study evaluated the feasibility of an automated Trueprep AUTO extraction with minimal biocontainment requirement from a direct sputum sample for performing tNGS with a portable MinION device (ONT). As reported in previous studies, the tNGS runs showed good depth, coverage, and SNP distribution (13–20). Previously, studies used different sample types, and in contrast to our study, all the samples were decontaminated inside the BSL3 facility routinely before proceeding to the DNA extraction (15, 17, 33, 34). Rapid diagnosis of DR-TB profile by tNGS is essential to ensure timely initiation of appropriate DR-TB regimen (2, 3).

The DNA extraction method is critical to achieve good-quality DNA for a successful tNGS run. Since sputum samples are mucopurulent, proper lysis and liquefaction procedures are required. Many automated instruments and kits with liquid handling systems are available for high-throughput DNA extraction from sputum samples in tNGS (7). Typically, these methods use a mechanical bead beating or chemical lysis, followed by silica spin columns or magnetic bead-based purification of DNA, commonly performed inside a BSL3 or TB containment facility (7). Previous studies have reported using direct sputum with the Deeplex Myc-TB kit in the tNGS (14, 15, 17).

In this study, two DNA extraction methods from direct or processed sputum were compared, both of which are part of routine TB diagnosis in LMICs, ensuring no additional costs of the extraction kit. The Trueprep AUTO does not require decontamination of the sample inside a BSL3 laboratory, though the second method using Genolyse requires decontamination of sputum and a BSL3 facility for DNA extraction. Apart from sputum, cultures were used with the Trueprep AUTO method for MGIT cultures compared to DNA extracted by the cTAB method from solid LJ medium. The distribution and median concentration for the Trueprep and Genolyse extraction methods from sputum show comparable yields. The quantity and quality were adequate for the PCR-based tNGS procedure. In a previous study on automated extraction instruments, the Trueprep AUTO device was recommended for DNA extraction for sequencing purposes (35).

As a LMIC setting with a high TB burden, 3,615 Truenat machines were positioned by the Government of India in 2022 at the district and subdistrict levels (36). Therefore, looking at the feasibility of using Trueprep DNA for tNGS is encouraging. Trueprep AUTO device is an automated, battery-operated DNA extraction device used in POCT using Truenat MTB and MTB plus. Trueprep AUTO yields around 100 μL of good-quality DNA from which only 6–12 μL is used for testing MTB-RIF. Therefore, using the remaining Trueprep DNA for alternative direct molecular testing like LPA is feasible, as reported earlier (37–39). At a CFU/mL of 10^5^–10^8^, Trueprep DNA yielded valid test results for line probe assays, mitigating the requirement of additional sample processing and DNA extraction inside a BSL3 laboratory (38, 39). Hence, the use of the Trueprep AUTO device does not require sample decontamination inside BSL3, and the DNA extraction can be done in a peripheral setting without sophistication. The system works with a simple liquefaction and lysis before DNA extraction from direct sputum without the requirement of sputum processing. It could be used for the follow-on tNGS testing in a programmatic setting.

In our study, Trueprep AUTO yielded less DNA with MGIT cultures, possibly due to the low input volume of MGIT cultures, and can be optimized when DNA extraction from MGIT cultures is required.

Previous studies with the Deeplex Myc-TB kit and in-house primers have shown successful tNGS runs with MinION and Oxford Nanopore Technology, comparable to the Illumina platform (14, 34, 40, 41). Genescreen offers an online bioinformatic tool compatible with Deeplex Myc-TB kits that can be used with Illumina platforms. Illumina platforms generate paired-end reads, producing FASTQ files for forward and reverse reads. The output with tNGS using rapid barcoding yields multiple reads, which the pipeline provided by Genoscreen does not support. Thus, this study used a customized bioinformatic pipeline for lineage prediction and variant calling as previously done for Deeplex Myc-TB when used with the MinION system (14).

In our study, the predominant direct sputum sample types were smear-positive samples graded 2+ and/or CFU/mL of 10^5^ or 10^6^, indicating a higher bacillary load. tNGS results are validated primarily based on each run’s experimental setup, as the samples have a higher bacillary load. Previous studies with tNGS on direct sputum showed better results with greater bacillary load, identified as higher smear grade or CFUs/cTs on baseline PCRs as part of the diagnosis (15, 34, 42). For smear-negative or low bacillary load samples like pediatric or HIV co-infected patients, tNGS could be performed using a more optimized protocol with Trueprep AUTO system on MGIT cultures. The turnaround time for tNGS using direct sputum samples is 2–3 days, while culturing the low bacillary load samples with MGIT would take an additional 3–4 weeks. All previous studies with Deeplex Myc-TB, except one that used the MinION, have used the Deeplex Genoscreen for bioinformatic analysis to predict lineage and DR in *M. tb* isolates (13, 15–20). Our customized pipeline successfully predicted lineage and SNPs with good concordance to LPA and WGS results across the first-line, second-line, and newer and repurposed drugs. The depth of genes covered by MinION showed variability and a heterogeneous distribution, observed by the differences in the depth calculated as mean and interquartile range (IQR). Poor representation of a few genes, like *rrl* and *rrs,* was observed, similar to the previous study with MinION using Deeplex Myc-TB primers (14).

Our study showed a predominance of Lineage 1 among the tested samples; it should be noted that most of the patients in our study were from South India. Previous molecular epidemiology studies showed that Lineage 1 was the most prevalent *M. tb* genotype in samples obtained from South Indian patients with pulmonary TB (43–45). Thus, our findings are consistent with the observations reported in previous epidemiological studies in India.

Analysis of the DR profile from our study identified the distribution of resistance to first- and second-line drugs. While the sensitivity and specificity obtained for RIF and INH were comparable to previous reports by WHO, performance with FQ showed far more superiority in our study than earlier (2). The performance characteristics in terms of sensitivity, specificity, and concordance were 100% with LZD in our study, with Cohen’s kappa agreement of 1.00. Due to the absence of any BDQ and CFZ resistance, it was not possible to compare with the previous reports, though the test showed a specificity of 100% (2). We recommend the inclusion of more LZD-, BDQ-, and CFZ-resistant samples to understand the performance of tNGS for these drugs. Discordance between LPA and tNGS for RIF and AMG was identified as regions outside the coverage of LPA. As recommended by WHO discordance resolution, the result obtained with tNGS was considered final (2). Among the *gyrB* and *gidB* genes showing additional resistance, WGS and pDST showed concordance, and DR prediction was done using the WGS and pDST results as recommended (2). For the missing *katG* gene in tNGS, pDST showed INH sensitivity and discordance resolved on comparison with the reference standard. Among the discordances observed in contrast to pDST, PZA showed similar discordance with tNGS and WGS. The pDST result for PZA was taken as conclusive for DR detection, considering the diversity of the SNPs observed for PZA (Supplementary Fig. 3).

Among the newer drugs, two LZD-resistant strains also carried resistance to BDQ and CFZ but were not picked up in tNGS. With WGS results, the resistance to BDQ and CFZ was analyzed and found to carry Rv1979C (A-129G), which is not included in the primers available in the Deeplex Myc-TB kit. Mutations in Rv1979c, a permease, have been shown to result in resistance to BDQ and CFZ less frequently than Rv0687 (46–48). Many of the previous NGS studies performed with in-house primers included these primers among others for detecting first- and second-line drugs and newer and repurposed drugs (33–35, 40–42, 49). Alternatively, *M. tb* genes implicated in BDQ, DLM, LZD, and CFZ resistance were tested with in-house primers in a single kit/reaction (50). As the commercial Deeplex Myc-TB kit lacks a few additional genes to detect BDQ and CFZ, adding primer pairs to detect DR to these drugs in tNGS from direct sputum would greatly benefit the rapid DR profiling of samples.

**Fig 8:**
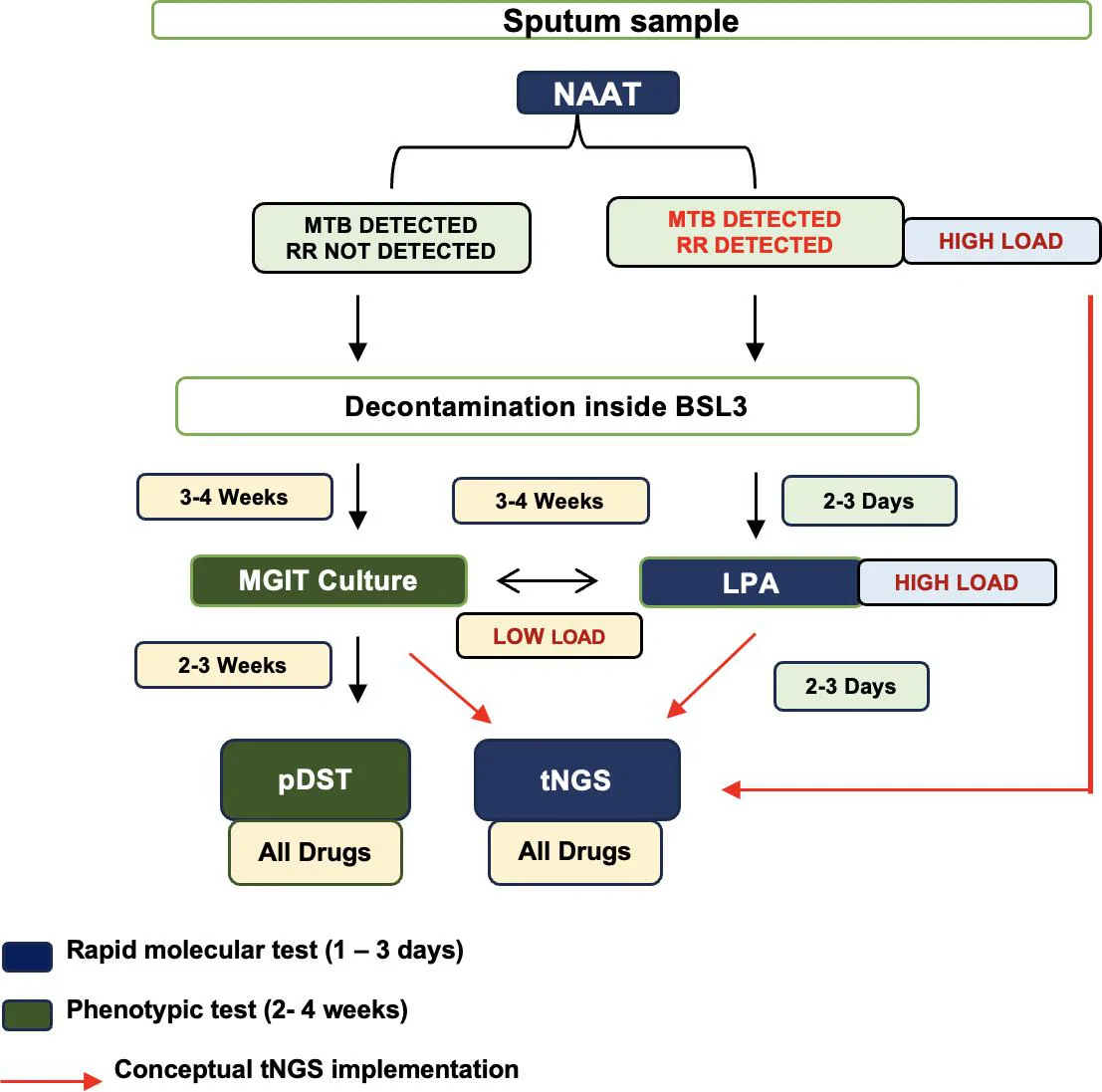
Conceptual diagnostic algorithm for tNGS in the low- and moderate-level TB testing laboratory using compact, low-cost Oxford Nanopore Technologies. tNGS can be performed at different tiers of TB laboratories upon detection of DR-TB. TB—Tuberculosis; MTB*—Mycobacterium tuberculosis*; NAAT—nucleic acid amplification technology; RR—rifampicin resistant; BSL3—Biosafety level 3 laboratory; LPA—line probe assay; MGIT—mycobacterial growth indicator tube; tNGS—targeted next-generation sequencing; pDST—phenotypic drug susceptibility testing.

The implementation of tNGS from direct sputum could have added diagnostic value when used as a follow-on test for additional DR. We propose a new conceptual diagnostic algorithm that includes tNGS in the low, moderate, and high-level TB testing laboratory using a compact, low-cost ONT device (Fig. 8). In the routine TB testing algorithm, the low-level labs perform NAAT and/or smear microscopy to detect *M. tb*. In this study, we recommend that upon detection of any resistance by NAAT or LPA, tNGS could be performed on direct sputum samples with higher bacillary load using the Trueprep DNA. Alternatively, MGIT culture could be used to perform tNGS and pDST for low-load bacillary samples. While using Trueprep DNA from direct sputum, the initiation of tNGS is feasible within 24 hours without the need for a biocontainment facility for DNA extraction, followed by 2–3 days for the actual tNGS run. Typically, for a low bacillary load sample, Trueprep AUTO extraction could be optimized for use with MGIT culture under the programmatic setting for tNGS. When tNGS is performed from MGIT culture, it would take 2–3 weeks for culture positivity. Furthermore, there is a requirement of biocontainment for DNA extraction followed by 2–3 days per run but still more rapid than pDST which could take another 2–3 weeks (Fig. 8). With the introduction of the 6–9 month BPaLM regimen (6–9 months), rapid comprehensive DR profiling would be an advantage considering shorter duration of therapy. In a previous study, a budget impact assessment was performed to evaluate the cost-effectiveness of tNGS in DR-TB detection in LMICs like Georgia, South Africa, and India by developing a stochastic decision analysis model (2, 51). The bacteriological confirmation is done by Xpert MTB/RIF in all three countries. The follow-on test is performed using Xpert XDR and pDST in South Africa and Georgia, while LPA and pDST are used in India. To estimate the incremental cost-effectiveness of tNGS after an initial molecular test for bacteriological confirmation, a decision analysis modeling approach was used. In countries like Georgia and South Africa, the introduction of tNGS would be more expensive than LPA plus pDST. But, in India, implementation of tNGS showed an approximate annual cost of US$ 57,130,727, which is less than US$ 57,719,097 for LPA followed by pDST (2, 51). In LMIC settings, the number of labs testing for pDST of Group A drugs like BDQ and LZD would be limited, thus missing out on patients for complete DR-TB testing, indicating an added advantage of tNGS over pDST.

Apart from the cost, other concerns over the feasibility of tNGS as a decentralized test included startup challenges in terms of infrastructure, technical complexity of the test, choice of platforms, requirement of skilled personnel, uninterrupted power supply, data management, supply chain, and timely updation of mutation catalog (2, 52). In a country like India, there is a fairly robust LPA testing with a facility for decontamination of samples, a laboratory setup, trained laboratory technicians for multiplex PCR, and sound knowledge of quality assurance for molecular testing (7). Considering the requirement of rapid DR-TB diagnostics, this pilot study warrants further multicentric validation to confirm the sensitivity, specificity, and operational feasibility of decentralizing tNGS and further use of the bioinformatic pipeline under the NTEP setting.

The study’s limitations include the lack of testing of rigorous statistical analysis of paired sputum and culture samples in the run and bioinformatic parameters, smear-negative NAAT-positive samples, the testing of different samples across different methods of DNA extraction rather than using the same sample as sputum, processed deposit, and cultures, and the unavailability of a complete DR profile for newer drugs like BDQ, CFZ, DLM, and PTM due to the lack of primer pairs for these genes in the commercial kit used in this study. Reproducibility of testing using duplicate samples was not calculated in this study.

We propose an extension of this pilot evaluation into a multicentric study involving more peripheral labs for establishing the sensitivity and specificity of this testing across different tiers of TB laboratories.

### Conclusions

Diagnosis of TB with bacteriological confirmation, followed by rapid comprehensive DR profiling, offers a challenge to DR-TB management globally (53). With the availability of WHO-endorsed low and moderate complexity NAATs, initial and follow-up diagnosis of TB for detection of resistance to first- and second-line drugs has become feasible. DR profile for other TB drugs is challenging, and direct tNGS could be an opportunity to achieve the same (7). Since newer and repurposed drugs have been integrated into the DR-TB regimens, it is essential to test their resistance profile in patient samples earlier to pDST so that adequate and appropriate treatment can be initiated (2, 3).

Our pilot evaluation study indicated the usefulness of DNA extraction from direct sputum or MGIT culture using a Trueprep AUTO extraction device, independent of a BSL3 or TB containment lab, under the programmatic setting. This approach would help treat MDR and XDR cases effectively and reduce the population’s generation and transmission of DR *M. tb* strains. Overall, this study promises to use DNA extracted under programmatic settings and rapid DR testing using a compact ONT device for tNGS with minimal biocontainment wherever possible. The bioinformatic analysis could be done at a higher laboratory or locally, based on the availability of resources and facilities at the testing site. A multicentric evaluation with a decentralized setup would offer better inputs on the operational feasibility of tNGS to ensure equity in a rapid and extensive DR-TB testing under the programmatic setting.

## Data Availability

Data available publicly at NCBI Sequence Read Archive with the following ID. Accession: PRJNA1177198 ID: 1177198. The in-house tNGS pipeline has been provided in the following link (https://github.com/shanmugamsiva/tNGS).

## SUPPLEMENTAL MATERIAL

Supplemental material spectrum.02917-25-s0001.docx Fig. S1 to S3; Tables S1 and S2.

ASM does not own the copyrights to Supplemental Material that may be linked to, or accessed through, an article. The authors have granted ASM a non-exclusive, world-wide license to publish the Supplemental Material files. Please contact the corresponding author directly for reuse.
