# Supplementary material for: Pilot evaluation of a compact targeted next-generation sequencing with minimum biocontainment for rapid diagnosis of drug-resistant tuberculosis
Source: Microbiol Spectr. 2026 May 18;14(7):e02917-25. doi: 10.1128/spectrum.02917-25 (PMC13340021; doi:10.1128/spectrum.02917-25)
Supplement: Supplemental material — Fig. S1 to S3; Tables S1 and S2. [file spectrum.02917-25-s0001.docx]

**FIG S1**

**Supplementary Fig 1 -** Smear grades of direct sputum samples extracted using Genolyse and Trueprep with their success in tNGS runs. The X-axis indicates the different smear grades and Y-axis indicates the number of samples.

**FIG S2**

**Supplementary Fig 2 -** Bacillary load of direct sputum samples as estimated by CFUs/ml in Trueprep and their success in tNGS runs. The X-axis indicates the log10 values of CFUs/ml and Y-axis indicates the number of samples.

**FIG S3**

**Supplementary Fig 3 –** Diversity of single nucleotide polymorphisms (SNPs) observed among the first line and second line drugs among samples identified by tNGS in this study. The figure shows the distribution of SNPs among (A) *rpoB* of RIF; (B) *katG, kasA* and *inhA* of INH; (C) *embB* of EMB; (D) *pncA* of PZA; (E) *gyrA* and *gyrB* of FQ; (F) *rrs, rrl, rplC*, *gid* and *eis* promoter of AMG

**TABLE S1**

| Depth (IQR) | | | |
| --- | --- | --- | --- |
|  | **Mean** | **SD** | **CV** |
| *rpoB* | 331.85 | 138.30 | 0.42 |
| *katG* | 101.84 | 39.65 | 0.39 |
| *inhA* | 693.56 | 405.14 | 0.58 |
| *ahpC* | 197.97 | 79.96 | 0.40 |
| *fabG1* | 207.16 | 83.72 | 0.40 |
| *ethA* | 102.67 | 41.05 | 0.40 |
| *gyrA* | 84.74 | 28.70 | 0.34 |
| *gyrB* | 162.85 | 70.96 | 0.44 |
| *rrs* | 54.96 | 20.17 | 0.37 |
| *eis* | 190.28 | 106.10 | 0.56 |
| *embB* | 91.69 | 34.72 | 0.38 |
| *pncA* | 495.69 | 208.07 | 0.42 |
| *rpsl* | 222.65 | 93.68 | 0.42 |
| *gid* | 264.90 | 111.11 | 0.42 |
| *tlyA* | 114.37 | 47.97 | 0.42 |
| *rrl* | 46.17 | 15.55 | 0.34 |
| *rplC* | 236.47 | 101.53 | 0.43 |
| *Rv0678* | 437.56 | 235.83 | 0.54 |
| *hsp65* | 188.56 | 78.72 | 0.41 |

**Supplementary Table 1:** The depth of diffeent genes obtained in the study are presented as mean with SD and CV with its IQR.

IQR – Interquartile range; SD – Standard deviation; CV – Coefficient of variation

**Table S2**

| Coverage (in percentage) | | | |
| --- | --- | --- | --- |
|  | **Mean** | **SD** | **CV** |
| *rpoB* | 90.67 | 13.08 | 0.14 |
| *katG* | 91.08 | 12.46 | 0.14 |
| *inhA* | 76.33 | 0.65 | 0.01 |
| *ahpC* | 78.21 | 14.32 | 0.18 |
| *fabG1* | 89.04 | 12.64 | 0.14 |
| *ethA* | 98.95 | 4.91 | 0.05 |
| *gyrA* | 94.71 | 9.25 | 0.10 |
| *gyrB* | 91.48 | 12.82 | 0.14 |
| *rrs* | 99.99 | 0.08 | 0.00 |
| *eis* | 95.00 | 9.46 | 0.10 |
| *embB* | 93.06 | 9.21 | 0.10 |
| *pncA* | 99.99 | 0.06 | 0.00 |
| *rpsl* | 100.00 | 0.00 | 0.00 |
| *gid* | 98.95 | 1.02 | 0.01 |
| *tlyA* | 100.00 | 0.00 | 0.00 |
| *rrl* | 95.15 | 10.61 | 0.11 |
| *rplC* | 88.79 | 14.10 | 0.16 |
| *Rv0678* | 100.00 | 0.00 | 0.00 |
| *hsp65* | 79.07 | 14.71 | 0.19 |

**Supplementary Table 2:** The coverage of diffeent genes (in percentage) obtained in the study are presented as mean with SD and CV.

SD – Standard deviation; CV – Coefficient of variation
